# Supplementary material for: Co-factor-free aggregation of tau into seeding-competent RNA-sequestering amyloid fibrils
Source: Nat Commun. 2021 Jul 9;12:4231. doi: 10.1038/s41467-021-24362-8 (PMC8270918; doi:10.1038/s41467-021-24362-8)
Supplement: Supplementary file 1 — Supplementary Information [file 41467_2021_24362_MOESM1_ESM.pdf]

Supplementary information for

Co-factor-free aggregation of tau into seeding-competent RNA-sequestering amyloid fibrils

Pijush Chakraborty, Gwladys Rivière, Shu Liu, Alain Ibáñez de Opakua, Riza Dervişoğlu, Alina Hebestreit, Loren B. Andreas, Ina M. Vorberg and Markus Zweckstetter

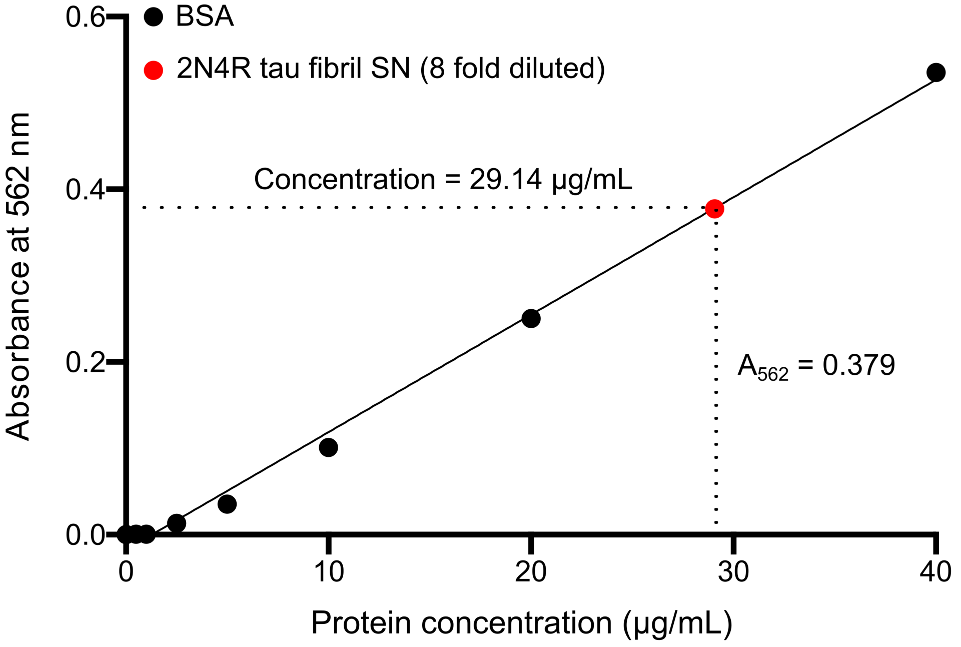

Concentration of 2N4R tau before aggregation = 1.146 mg/mL

Concentration of 2N4R tau in SN = (29.14 x 8) µg/mL = 0.233 mg/mL

~ 80 % 2N4R tau aggregated

Supplementary Fig. 1 | Determination of the concentration of 2N4R tau present in the supernatant (SN; red dot; 8-fold dilution) after aggregation in the absence of heparin. Concentrations were determined using the BCA assay. Absorbance values, which were detected for BSA standard solutions with known concentrations, are shown in black.

Sequence	Observed m/z	Absolute mass (Da)	Charge	TIC
LQTAPVPMPDLK	663.3665	1,324.72	2	1085120
LQTAPVPMPDLK	663.3668	1,324.72	2	488941
IGSTENLK	431.2404	860.4663	2	8.25E+07
IGSTENLK	431.2383	860.4621	2	4.33E+07
IGSTENLK	431.2396	860.4646	2	3.77E+07
IGSTENLK	431.2394	860.4643	2	7759340
IGSTENLK	431.2393	860.464	2	1.84E+07
IGSTENLK	431.2398	860.4651	2	5.58E+07
IGSTENLK	431.2397	860.4648	2	370087
IGSTENLK	431.2382	860.4618	2	899899
IGSTENLK	431.2389	860.4632	2	623917
IGSTENLK	431.2384	860.4623	2	451482
IGSTENLK	431.2404	860.4663	2	380415
IGSTENLK	431.2386	860.4627	2	846224
IGSTENLKHQPGGGK	508.2739	1,521.80	3	259432
IGSTENLKHQPGGGK	381.4566	1,521.80	4	184486
HQPGGGKVQIINK	459.2663	1,374.78	3	4335000
HQPGGGKVQIINK	459.2674	1,374.78	3	887532
HQPGGGKVQIINK	459.2677	1,374.78	3	490639
HQPGGGKVQIINK	459.2662	1,374.78	3	712940
HQPGGGKVQIINK	459.2674	1,374.78	3	397008
VQIINKK	421.7779	841.5412	2	1974720
VQIINKK	421.7784	841.5423	2	2.86E+07
VQIINKK	421.7774	841.5402	2	5661730
VQIINKK	421.7774	841.5402	2	2975050
VQIINKK	421.7784	841.5423	2	517547
VQIINKK	421.779	841.5434	2	2.54E+07
VQIINKK	421.7782	841.5419	2	1.11E+07
VQIINKK	421.7787	841.5428	2	2.52E+07
VQIINKK	421.7789	841.5432	2	881426
VQIINKK	421.7775	841.5404	2	2254640
VQIINKK	421.7777	841.5408	2	2.51E+07
VQIINKK	421.7781	841.5415	2	2.88E+07
VQIINKK	421.7788	841.5431	2	1766970
VQIINKK	421.778	841.5414	2	1.80E+07
VQIINKK	421.7773	841.5401	2	5624820
VQIINKK	421.7789	841.5432	2	3.11E+07
VQIINKK	421.778	841.5415	2	1221890
VQIINKK	421.7782	841.5418	2	423916
VQIINKK	421.7779	841.5412	2	447356
VQIINKK	421.7775	841.5404	2	459569
VQIINKK	421.7788	841.5431	2	536818
VQIINKKLDLSNVQSK	457.5269	1,826.08	4	623597
QIINKK	372.2438	742.4731	2	1779580
QIINKK	372.2432	742.4719	2	1993940
KLDLSNVQ	458.7625	915.5105	2	1086550
KLDLSNVQSK	566.3255	1,130.64	2	1.44E+07
KLDLSNVQSK	566.3273	1,130.64	2	3897200
KLDLSNVQSK	566.326	1,130.64	2	7390680
KLDLSNVQSK	566.3244	1,130.63	2	9.73E+07
KLDLSNVQSK	566.3249	1,130.64	2	1.12E+08
KLDLSNVQSK	566.3258	1,130.64	2	1482140
KLDLSNVQSK	566.3266	1,130.64	2	1472020
KLDLSNVQSK	566.323	1,130.63	2	4895010
KLDLSNVQSK	566.3239	1,130.63	2	1.53E+07
KLDLSNVQSK	566.3268	1,130.64	2	996572
KLDLSNVQSK	566.3272	1,130.64	2	1049920
KLDLSNVQSK	566.3276	1,130.64	2	5839370
KLDLSNVQSK	566.324	1,130.63	2	3.11E+07
KLDLSNVQSK	566.3257	1,130.64	2	1630260
KLDLSNVQSK	566.3244	1,130.63	2	1.17E+07
KLDLSNVQSK	566.327	1,130.64	2	621179
KLDLSNVQSK	566.3265	1,130.64	2	1641550
KLDLSNVQSK	566.3248	1,130.64	2	7.11E+07
KLDLSNVQSK	566.3248	1,130.64	2	3.45E+07
KLDLSNVQSK	566.3266	1,130.64	2	2668430

Sequence	Observed m/z	Absolute mass (Da)	Charge	TIC
KLDLSNVQSK	566.325	1,130.64	2	688486
KLDLSNVQSK	377.8875	1,130.64	3	1543860
KLDLSNVQSK	566.3237	1,130.63	2	1.02E+07
KLDLSNVQSK	377.8856	1,130.63	3	1.48E+07
KLDLSNVQSK	377.8873	1,130.64	3	1270980
KLDLSNVQSK	377.8866	1,130.64	3	2090420
KLDLSNVQSK	377.8857	1,130.64	3	8531420
KLDLSNVQSK	566.3245	1,130.63	2	564889
KLDLSNVQSK	377.887	1,130.64	3	1.03E+08
KLDLSNVQSK	566.3256	1,130.64	2	424992
KLDLSNVQSK	377.8865	1,130.64	3	1810990
KLDLSNVQSK	377.8867	1,130.64	3	5202380
KLDLSNVQSK	566.3248	1,130.64	2	389941
KLDLSNVQSK	566.3263	1,130.64	2	2100210
KLDLSNVQSK	377.8846	1,130.63	3	3.19E+07
KLDLSNVQSK	377.8861	1,130.64	3	2383330
KLDLSNVQSK	377.8848	1,130.63	3	1.29E+08
KLDLSNVQSK	377.8873	1,130.64	3	3748570
KLDLSNVQSK	377.8847	1,130.63	3	2.92E+07
KLDLSNVQSK	566.3254	1,130.64	2	398022
KLDLSNVQSK	377.8855	1,130.63	3	8.94E+07
KLDLSNVQSK	377.8847	1,130.63	3	787084
KLDLSNVQSK	377.885	1,130.63	3	3073160
KLDLSNVQSK	377.8874	1,130.64	3	4185900
KLDLSNVQSK	377.8843	1,130.63	3	9419510
KLDLSNVQSK	377.8844	1,130.63	3	8.83E+07
KLDLSNVQSK	377.8846	1,130.63	3	1.60E+07
KLDLSNVQSK	377.8857	1,130.64	3	1774280
KLDLSNVQSK	377.886	1,130.64	3	1.05E+07
KLDLSNVQSK	377.8846	1,130.63	3	1374120
KLDLSNVQSK	566.3251	1,130.64	2	357209
KLDLSNVQSK	566.3255	1,130.64	2	245725
KLDLSNVQSK	566.3232	1,130.63	2	389543
KLDLSNVQSK	377.8852	1,130.63	3	1125230
KLDLSNVQSK	566.3254	1,130.64	2	234322
KLDLSNVQSK	566.3273	1,130.64	2	442197
KLDLSNVQSK	566.3274	1,130.64	2	240791
LDLSNVQSK	502.2765	1,002.54	2	1.10E+08
LDLSNVQSK	502.2768	1,002.54	2	2.53E+07
LDLSNVQSK	502.2781	1,002.54	2	2.82E+07
LDLSNVQSK	502.2783	1,002.54	2	1.07E+07
LDLSNVQSK	502.2776	1,002.54	2	534743
LDLSNVQSK	502.2746	1,002.53	2	3000260
LDLSNVQSK	502.2748	1,002.54	2	3.65E+07
LDLSNVQSK	502.2784	1,002.54	2	5150750
LDLSNVQSK	502.2776	1,002.54	2	708110
LDLSNVQSK	502.2777	1,002.54	2	1712560
LDLSNVQSK	502.2783	1,002.54	2	2509220
LDLSNVQSK	502.2787	1,002.54	2	777637
LDLSNVQSK	502.2791	1,002.54	2	639433
LDLSNVQSK	502.2792	1,002.54	2	1122120
LDLSNVQSK	502.2781	1,002.54	2	328470
LDLSNVQSK	502.2778	1,002.54	2	345601
LDLSNVQSK	502.2767	1,002.54	2	951925
LDLSNVQSK	502.2763	1,002.54	2	407844
CGSKDNIK	461.229	920.4434	2	1325970
CGSKDNIK	461.2292	920.4438	2	1558090
CGSKDNIK	461.2301	920.4455	2	1260580
CGSKDNIK	461.2299	920.4452	2	1359890
CGSKDNIK	461.229	920.4434	2	928951
DNIKHVPGGGSVQIVYKPVDLSK	613.3406	2,449.33	4	402953
HVPGGGSVQ	419.2169	836.4192	2	5175300
HVPGGGSVQIVY	606.8246	1,211.63	2	5196730
HVPGGGSVQIVY	606.8227	1,211.63	2	1927530
HVPGGGSVQIVY	606.8281	1,211.64	2	607544
HVPGGGSVQIVY	606.8248	1,211.64	2	658417

Sequence	Observed m/z	Absolute mass (Da)	Charge	TIC
HVPGGGSVQIVYK	447.5846	1,339.73	3	3309080
HVPGGGSVQIVYK	447.5861	1,339.74	3	1744260
HVPGGGSVQIVYK	447.5854	1,339.73	3	2346670
HVPGGGSVQIVYK	670.8753	1,339.74	2	2555930
HVPGGGSVQIVYK	670.8753	1,339.74	2	1447930
HVPGGGSVQIVYK	670.873	1,339.73	2	2009850
HVPGGGSVQIVYKPVDL	588.9972	1,763.97	3	1244210
HVPGGGSVQIVYKPVDL	588.9971	1,763.97	3	1857990
HVPGGGSVQIVYKPVDLSK	660.7059	1,979.10	3	4.06E+07
HVPGGGSVQIVYKPVDLSK	990.5541	1,979.09	2	3740060
HVPGGGSVQIVYKPVDLSK	495.7817	1,979.10	4	9258010
HVPGGGSVQIVYKPVDLSK	990.557	1,979.10	2	6676660
HVPGGGSVQIVYKPVDLSK	495.7825	1,979.10	4	3428870
HVPGGGSVQIVYKPVDLSK	495.7829	1,979.10	4	2320600
HVPGGGSVQIVYKPVDLSK	495.783	1,979.10	4	2371390
HVPGGGSVQIVYKPVDLSK	660.7048	1,979.09	3	3.59E+08
HVPGGGSVQIVYKPVDLSK	495.7805	1,979.09	4	2914550
HVPGGGSVQIVYKPVDLSK	660.7063	1,979.10	3	9921910
HVPGGGSVQIVYKPVDLSK	495.783	1,979.10	4	5478000
HVPGGGSVQIVYKPVDLSK	495.7815	1,979.10	4	3192610
HVPGGGSVQIVYKPVDLSK	495.7822	1,979.10	4	4334490
HVPGGGSVQIVYKPVDLSK	495.7813	1,979.10	4	2.57E+07
HVPGGGSVQIVYKPVDLSK	660.7051	1,979.09	3	1.48E+08
HVPGGGSVQIVYKPVDLSK	495.7816	1,979.10	4	1.11E+07
HVPGGGSVQIVYKPVDLSK	495.7821	1,979.10	4	2.84E+07
HVPGGGSVQIVYKPVDLSK	495.7801	1,979.09	4	7.49E+07
HVPGGGSVQIVYKPVDLSK	660.7036	1,979.09	3	4.47E+08
HVPGGGSVQIVYKPVDLSK	495.78	1,979.09	4	2.57E+08
HVPGGGSVQIVYKPVDLSK	495.7807	1,979.09	4	6.33E+07
HVPGGGSVQIVYKPVDLSK	660.7083	1,979.10	3	1.23E+07
HVPGGGSVQIVYKPVDLSK	660.7052	1,979.09	3	1.43E+08
HVPGGGSVQIVYKPVDLSK	495.7815	1,979.10	4	2583980
HVPGGGSVQIVYKPVDLSK	990.5507	1,979.09	2	2008380
HVPGGGSVQIVYKPVDLSK	495.7827	1,979.10	4	2159700
HVPGGGSVQIVYKPVDLSK	495.7801	1,979.09	4	3.08E+08
HVPGGGSVQIVYKPVDLSK	990.5532	1,979.09	2	7801890
HVPGGGSVQIVYKPVDLSK	660.7071	1,979.10	3	1.17E+07
HVPGGGSVQIVYKPVDLSK	990.5562	1,979.10	2	381200
HVPGGGSVQIVYKPVDLSK	660.7081	1,979.10	3	1.13E+07
HVPGGGSVQIVYKPVDLSK	660.7067	1,979.10	3	1.17E+07
HVPGGGSVQIVYKPVDLSK	660.7082	1,979.10	3	3.74E+07
HVPGGGSVQIVYKPVDLSK	495.7822	1,979.10	4	2446550
HVPGGGSVQIVYKPVDLSK	660.7058	1,979.10	3	1.38E+07
HVPGGGSVQIVYKPVDLSK	660.7045	1,979.09	3	2311750
GGGSVQIVYKPVDLSK	549.6429	1,645.91	3	945519
GGSVQIVYKPVDLSK	530.6381	1,588.89	3	1860150
GGSVQIVYKPVDLSK	530.6366	1,588.89	3	2202040
GGSVQIVYKPVDLSK	511.6294	1,531.87	3	3717640
GGSVQIVYKPVDLSK	511.6291	1,531.87	3	1347090
SVQIVYKPVDLSK	492.6227	1,474.85	3	1216430
SVQIVYKPVDLSK	738.4289	1,474.84	2	815127
SVQIVYKPVDLSK	738.4314	1,474.85	2	979506
SVQIVYKPVDLSK	492.6235	1,474.85	3	1558740
IVYKPVDLSK	387.9027	1,160.69	3	840122
IVYKPVDLSK	387.903	1,160.69	3	922994
KPVDLSK	393.7404	785.4662	2	555221
KPVDLSK	393.7412	785.4678	2	387968
KPVDLSK	393.7415	785.4685	2	568540
KPVDLSK	393.74	785.4654	2	591744
KPVDLSK	393.7413	785.468	2	430025
KPVDLSK	393.7404	785.4663	2	579873
KPVDLSK	393.7396	785.4647	2	364953
KPVDLSK	393.7407	785.4668	2	466148
KPVDLSK	393.7422	785.4699	2	339201
KPVDLSK	393.7424	785.4702	2	816433
CGSLGNIHHK	374.8554	1,121.54	3	738854
CGSLGNIHHK	374.8553	1,121.54	3	889273
CGSLGNIHHKPGGGQVEVK	658.672	1,972.99	3	1137040
CGSLGNIHHKPGGGQVEVK	658.6739	1,973.00	3	1327920
CGSLGNIHHKPGGGQVEVK	658.6737	1,973.00	3	992953
CGSLGNIHHKPGGGQVEVK	494.2573	1,973.00	4	3370610
CGSLGNIHHKPGGGQVEVK	658.6732	1,973.00	3	1395720
CGSLGNIHHKPGGGQVEVK	658.6652	1,972.97	3	1190500
CGSLGNIHHKPGGGQVEVK	658.6742	1,973.00	3	1297690
CGSLGNIHHKPGGGQVEVK	658.6736	1,973.00	3	1622510
CGSLGNIHHKPGGGQVEVK	658.6757	1,973.01	3	269325
CGSLGNIHHKPGGGQVEVK	494.2581	1,973.00	4	2476020
CGSLGNIHHKPGGGQVEVK	658.6744	1,973.00	3	374600
CGSLGNIHHKPGGGQVEVK	658.6737	1,973.00	3	932126
CGSLGNIHHKPGGGQVEVK	494.2572	1,973.00	4	4194500
CGSLGNIHHKPGGGQVEVK	494.257	1,973.00	4	3819660
CGSLGNIHHKPGGGQVEVK	658.6749	1,973.00	3	498095
CGSLGNIHHKPGGGQVEVK	658.6703	1,972.99	3	1691620
CGSLGNIHHKPGGGQVEVK	494.2565	1,973.00	4	2067020
CGSLGNIHHKPGGGQVEVK	494.2576	1,973.00	4	1971720
CGSLGNIHHKPGGGQVEVK	494.2567	1,973.00	4	631635
CGSLGNIHHKPGGGQVEVK	494.2575	1,973.00	4	3016150
CGSLGNIHHKPGGGQVEVK	494.2559	1,972.99	4	2382460
CGSLGNIHHKPGGGQVEVK	494.2588	1,973.01	4	1049420
CGSLGNIHHKPGGGQVEVK	494.2595	1,973.01	4	1020780
CGSLGNIHHKPGGGQVEVK	658.6698	1,972.99	3	499499
CGSLGNIHHKPGGGQVEVK	494.2555	1,972.99	4	843446
HKPGGGQVEVK	379.2153	1,134.62	3	788203
HKPGGGQVEVK	379.213	1,134.62	3	943892
KPGGGQVEVK	499.788	997.5615	2	320523
KPGGGQVEVK	499.7853	997.5561	2	93,917.00
PGGGQVEVK	435.7387	869.4629	2	675391
PGGGQVEVK	435.7404	869.4663	2	1.46E+07
PGGGQVEVK	435.7382	869.4619	2	1262160
PGGGQVEVK	435.7381	869.4617	2	2111660
PGGGQVEVK	435.7405	869.4665	2	2.70E+07
PGGGQVEVK	435.7384	869.4622	2	4518720
PGGGQVEVK	435.739	869.4635	2	1.56E+07
PGGGQVEVK	435.7386	869.4627	2	835934
PGGGQVEVK	435.7368	869.459	2	9522350
PGGGQVEVK	435.742	869.4694	2	528237
GGGQVEVK	387.2131	772.4116	2	322668
GGQVEVK	358.7026	715.3906	2	595542
SEKLDFK	433.7355	865.4564	2	2037840
SEKLDFK	433.7361	865.4577	2	1644280
SEKLDFK	433.736	865.4574	2	2002940
SEKLDFK	433.7367	865.4588	2	782481
SEKLDFK	433.7353	865.456	2	1552130
SEKLDFK	433.7364	865.4582	2	713546
SEKLDFK	433.7361	865.4576	2	1239020
SEKLDFK	433.7363	865.4581	2	370496
SEKLDFK	433.7362	865.4579	2	499564
LDFKDR	397.2141	792.4137	2	1977410
LDFKDR	397.2155	792.4164	2	1013110
LDFKDR	397.2157	792.4168	2	836590
LDFKDR	397.2175	792.4204	2	1887930
LDFKDR	397.2141	792.4136	2	917648
IGSLDNITHVPGGGNK	526.9511	1,577.83	3	1608920
IGSLDNITHVPGGGNK	526.9508	1,577.83	3	1536100
IGSLDNITHVPGGGNK	526.951	1,577.83	3	489459
IGSLDNITHVPGGGNK	526.9509	1,577.83	3	689049
IGSLDNITHVPGGGNKK	569.6486	1,705.92	3	440431
IGSLDNITHVPGGGNKK	569.6498	1,705.93	3	420993
KIETHK	378.2239	754.4333	2	130320
KIETHK	378.2263	754.4381	2	125368
KIETHK	378.2264	754.4382	2	693732

Supplementary Fig. 2 | Details of the peptides detected (shown in Fig. 2c, black) by enzymatic digestion of the trypsin protected core (Fig. 2b) of heparin-induced 2N4R tau fibrils.

Sequence	Observed m/z	Absolute mass (Da)	Charge	TIC
AKTDHGAEIVYK	666.352	1,330.69	2	2112160
AKTDHGAEIVYK	666.3525	1,330.69	2	1441290
AKTDHGAEIVYK	444.5705	1,330.69	3	2.02E+07
AKTDHGAEIVYK	444.57	1,330.69	3	5533990
CGSLGNIHHKPGGGQVEVK	987.5019	1,972.99	2	458225
CGSLGNIHHKPGGGQVEVK	658.6707	1,972.99	3	2941340
CGSLGNIHHKPGGGQVEVK	494.2547	1,972.99	4	1580130
DNIKHVPGGGSVQIVYKPVDLSK	817.4449	2,449.31	3	2528420
DNIKHVPGGGSVQIVYKPVDLSK	613.3411	2,449.34	4	7021630
DNIKHVPGGGSVQIVYKPVDLSK	817.4438	2,449.31	3	1155600
DNIKHVPGGGSVQIVYKPVDLSK	613.3351	2,449.31	4	5540500
DRVQSKIGSLDNITHVPGGGNK	764.7397	2,291.20	3	3097320
DRVQSKIGSLDNITHVPGGGNK	459.2466	2,291.20	5	2628490
DRVQSKIGSLDNITHVPGGGNKK	484.8668	2,419.30	5	1873910
HQPGGGKVQIINK	459.2658	1,374.78	3	690270
HQPGGGKVQIINKK	376.7255	1,502.87	4	352294
HVPGGGSVQIVYKPVDLSK	990.5506	1,979.09	2	1.50E+07
HVPGGGSVQIVYKPVDLSK	660.704	1,979.09	3	8369030
HVPGGGSVQIVYKPVDLSK	660.7055	1,979.09	3	5284610
HVPGGGSVQIVYKPVDLSK	660.7059	1,979.10	3	7011870
HVPGGGSVQIVYKPVDLSK	660.7037	1,979.09	3	4.69E+07
HVPGGGSVQIVYKPVDLSK	495.7793	1,979.09	4	949283
HVPGGGSVQIVYKPVDLSK	660.7061	1,979.10	3	2224980
HVPGGGSVQIVYKPVDLSK	660.7016	1,979.08	3	1.06E+07
HVPGGGSVQIVYKPVDLSK	660.7066	1,979.10	3	3102850
HVPGGGSVQIVYKPVDLSK	495.7789	1,979.09	4	1561800
HVPGGGSVQIVYKPVDLSK	660.7066	1,979.10	3	2940350
HVPGGGSVQIVYKPVDLSK	660.7041	1,979.09	3	1.25E+07
HVPGGGSVQIVYKPVDLSK	495.7804	1,979.09	4	722816
HVPGGGSVQIVYKPVDLSK	660.7077	1,979.10	3	2105500
HVPGGGSVQIVYKPVDLSK	660.7056	1,979.09	3	3327480
HVPGGGSVQIVYKPVDLSK	660.7067	1,979.10	3	1646850
HVPGGGSVQIVYKPVDLSK	495.7806	1,979.09	4	1388860
HVPGGGSVQIVYKPVDLSK	660.7051	1,979.09	3	4058520
HVPGGGSVQIVYKPVDLSK	660.7028	1,979.09	3	5430780
HVPGGGSVQIVYKPVDLSK	495.7783	1,979.08	4	1.01E+09
HVPGGGSVQIVYKPVDLSK	495.7788	1,979.09	4	847724
HVPGGGSVQIVYKPVDLSK	660.7027	1,979.09	3	1.19E+07
HVPGGGSVQIVYKPVDLSK	990.5495	1,979.08	2	1409330
HVPGGGSVQIVYKPVDLSK	495.7795	1,979.09	4	1153680
HVPGGGSVQIVYKPVDLSK	495.7788	1,979.09	4	4.69E+07
HVPGGGSVQIVYKPVDLSK	660.7034	1,979.09	3	1.47E+08
HVPGGGSVQIVYKPVDLSK	660.7051	1,979.09	3	3208480
HVPGGGSVQIVYKPVDLSK	660.704	1,979.09	3	2.81E+07
HVPGGGSVQIVYKPVDLSK	495.7811	1,979.10	4	771167
HVPGGGSVQIVYKPVDLSK	660.7043	1,979.09	3	9691810
HVPGGGSVQIVYKPVDLSK	495.7798	1,979.09	4	693595
HVPGGGSVQIVYKPVDLSK	495.7801	1,979.09	4	976561
HVPGGGSVQIVYKPVDLSK	660.7021	1,979.08	3	8.28E+07
HVPGGGSVQIVYKPVDLSK	495.7785	1,979.08	4	4.53E+08
HVPGGGSVQIVYKPVDLSK	495.7791	1,979.09	4	1097450
HVPGGGSVQIVYKPVDLSK	660.7044	1,979.09	3	1.00E+07
HVPGGGSVQIVYKPVDLSK	990.5496	1,979.08	2	3.50E+08
HVPGGGSVQIVYKPVDLSK	660.707	1,979.10	3	2223410
HVPGGGSVQIVYKPVDLSK	495.7791	1,979.09	4	4140980
HVPGGGSVQIVYKPVDLSK	660.7019	1,979.08	3	1.89E+07
HVPGGGSVQIVYKPVDLSK	495.7818	1,979.10	4	693787
HVPGGGSVQIVYKPVDLSK	660.7065	1,979.10	3	2884480
HVPGGGSVQIVYKPVDLSK	660.7054	1,979.09	3	4776200
HVPGGGSVQIVYKPVDLSK	495.7782	1,979.08	4	2.81E+07
HVPGGGSVQIVYKPVDLSK	495.781	1,979.09	4	1361110
HVPGGGSVQIVYKPVDLSK	660.706	1,979.10	3	2744780
HVPGGGSVQIVYKPVDLSK	660.7045	1,979.09	3	9240940
HVPGGGSVQIVYKPVDLSK	495.7788	1,979.09	4	793429
HVPGGGSVQIVYKPVDLSK	660.7033	1,979.09	3	1.32E+07
HVPGGGSVQIVYKPVDLSK	495.7794	1,979.09	4	1.22E+07
HVPGGGSVQIVYKPVDLSK	495.7797	1,979.09	4	1596920
HVPGGGSVQIVYKPVDLSK	660.7037	1,979.09	3	4333350
HVPGGGSVQIVYKPVDLSK	990.5502	1,979.09	2	406341
HVPGGGSVQIVYKPVDLSK	660.7068	1,979.10	3	4017330
HVPGGGSVQIVYKPVDLSK	495.779	1,979.09	4	8845590
HVPGGGSVQIVYKPVDLSK	495.7822	1,979.10	4	594417
HVPGGGSVQIVYKPVDLSK	495.7813	1,979.10	4	658746

Sequence	Observed m/z	Absolute mass (Da)	Charge	TIC
HVPGGGSVQIVYKPVDLSK	660.7082	1,979.10	3	2811180
HVPGGGSVQIVYKPVDLSK	660.7073	1,979.10	3	1991730
HVPGGGSVQIVYKPVDLSK	660.7075	1,979.10	3	2627530
HVPGGGSVQIVYKPVDLSK	660.7018	1,979.08	3	4.21E+09
HVPGGGSVQIVYKPVDLSK	660.7077	1,979.10	3	2581060
HVPGGGSVQIVYKPVDLSK	495.7808	1,979.09	4	532325
HVPGGGSVQIVYKPVDLSK	660.7045	1,979.09	3	2597390
HVPGGGSVQIVYKPVDLSK	495.7801	1,979.09	4	636190
HVPGGGSVQIVYKPVDLSK	495.781	1,979.09	4	532791
HVPGGGSVQIVYKPVDLSK	495.7811	1,979.10	4	714262
HVPGGGSVQIVYKPVDLSK	495.7824	1,979.10	4	482556
HVPGGGSVQIVYKPVDLSK	660.7021	1,979.08	3	1.16E+09
HVPGGGSVQIVYKPVDLSK	495.7807	1,979.09	4	581133
HVPGGGSVQIVYKPVDLSK	495.7804	1,979.09	4	906451
HVPGGGSVQIVYKPVDLSK	990.553	1,979.09	2	501700
HVPGGGSVQIVYKPVDLSK	660.7021	1,979.08	3	2.84E+07
HVPGGGSVQIVYKPVDLSK	495.7809	1,979.09	4	639708
HVPGGGSVQIVYKPVDLSK	495.7788	1,979.09	4	2486670
HVPGGGSVQIVYKPVDLSKVTSK	599.5907	2,394.33	4	2.31E+07
HVPGGGSVQIVYKPVDLSKVTSK	799.1186	2,394.33	3	4130730
IETHKLTFRENAK	793.9355	1,585.86	2	2190220
IETHKLTFRENAK	529.6262	1,585.86	3	1.15E+07
IETHKLTFRENAKAK	596.0042	1,784.99	3	1762750
IGSLDNITHVPGGGNK	789.9158	1,577.82	2	3.73E+08
IGSLDNITHVPGGGNK	789.9163	1,577.82	2	3.21E+07
IGSLDNITHVPGGGNK	789.9152	1,577.82	2	9.96E+08
IGSLDNITHVPGGGNK	789.9168	1,577.82	2	810409
IGSLDNITHVPGGGNK	526.9493	1,577.83	3	1651830
IGSLDNITHVPGGGNK	789.9173	1,577.82	2	2651900
IGSLDNITHVPGGGNK	526.9488	1,577.82	3	2066100
IGSLDNITHVPGGGNK	526.9498	1,577.83	3	1975390
IGSLDNITHVPGGGNK	789.9175	1,577.82	2	5733840
IGSLDNITHVPGGGNK	526.9476	1,577.82	3	1.48E+07
IGSLDNITHVPGGGNK	526.9492	1,577.83	3	1.53E+08
IGSLDNITHVPGGGNK	526.9499	1,577.83	3	2.83E+09
IGSLDNITHVPGGGNK	526.9506	1,577.83	3	1707060
IGSLDNITHVPGGGNK	526.949	1,577.83	3	1876050
IGSLDNITHVPGGGNK	526.9502	1,577.83	3	1906910
IGSLDNITHVPGGGNK	526.9468	1,577.82	3	3882530
IGSLDNITHVPGGGNK	526.9481	1,577.82	3	7.70E+08
IGSLDNITHVPGGGNK	526.9464	1,577.82	3	2791160
IGSLDNITHVPGGGNK	526.9465	1,577.82	3	3411380
IGSLDNITHVPGGGNK	526.946	1,577.82	3	1529550
IGSLDNITHVPGGGNK	526.9482	1,577.82	3	1737920
IGSLDNITHVPGGGNK	526.9475	1,577.82	3	2552780
IGSLDNITHVPGGGNK	526.9481	1,577.82	3	3058160
IGSLDNITHVPGGGNK	526.9468	1,577.82	3	9277720
IGSLDNITHVPGGGNK	526.946	1,577.82	3	1.13E+07
IGSLDNITHVPGGGNK	526.9472	1,577.82	3	2636520
IGSLDNITHVPGGGNK	526.9487	1,577.82	3	2985840
IGSLDNITHVPGGGNK	526.9488	1,577.82	3	1773780
IGSLDNITHVPGGGNK	526.9481	1,577.82	3	2273800
IGSLDNITHVPGGGNK	789.9166	1,577.82	2	889692
IGSLDNITHVPGGGNK	526.9468	1,577.82	3	3380620
IGSLDNITHVPGGGNK	526.9499	1,577.83	3	1899070
IGSLDNITHVPGGGNK	526.9486	1,577.82	3	2689600
IGSLDNITHVPGGGNK	789.9147	1,577.81	2	2997430
IGSLDNITHVPGGGNK	526.9478	1,577.82	3	4.87E+07
IGSLDNITHVPGGGNK	526.9464	1,577.82	3	3526230
IGSLDNITHVPGGGNK	526.9468	1,577.82	3	6623530
IGSLDNITHVPGGGNK	526.948	1,577.82	3	2208650
IGSLDNITHVPGGGNK	526.9481	1,577.82	3	5383120
IGSLDNITHVPGGGNK	526.9467	1,577.82	3	4841190
IGSLDNITHVPGGGNK	526.9462	1,577.82	3	2.41E+07
IGSLDNITHVPGGGNK	526.948	1,577.82	3	1.15E+08
IGSLDNITHVPGGGNK	526.9489	1,577.82	3	1.03E+07
IGSLDNITHVPGGGNK	526.9501	1,577.83	3	1713510
IGSLDNITHVPGGGNK	526.9457	1,577.82	3	4650700
IGSLDNITHVPGGGNK	526.9487	1,577.82	3	1656710
IGSLDNITHVPGGGNK	789.9152	1,577.82	2	2125410
IGSLDNITHVPGGGNK	526.9503	1,577.83	3	1577080
IGSLDNITHVPGGGNK	526.9464	1,577.82	3	9456110
IGSLDNITHVPGGGNK	526.95	1,577.83	3	1738300

Sequence	Observed m/z	Absolute mass (Da)	Charge	TIC
IGSLDNITHVPGGGNK	526.9491	1,577.83	3	1155100
IGSLDNITHVPGGGNK	526.9484	1,577.82	3	221418
IGSLDNITHVPGGGNK	526.9493	1,577.83	3	286334
IGSLDNITHVPGGGNK	526.9454	1,577.81	3	5477080
IGSLDNITHVPGGGNK	526.9497	1,577.83	3	2062070
IGSLDNITHVPGGGNK	526.9471	1,577.82	3	2340520
IGSLDNITHVPGGGNK	526.9483	1,577.82	3	208352
IGSLDNITHVPGGGNK	789.9179	1,577.82	2	1253410
IGSLDNITHVPGGGNK	789.9111	1,577.81	2	1343070
IGSLDNITHVPGGGNK	526.9494	1,577.83	3	166880
IGSLDNITHVPGGGNK	526.9495	1,577.83	3	321867
IGSLDNITHVPGGGNKK	853.964	1,705.91	2	1.66E+07
IGSLDNITHVPGGGNKK	853.9636	1,705.91	2	3424590
IGSLDNITHVPGGGNKK	569.6451	1,705.91	3	1.05E+08
IGSLDNITHVPGGGNKK	569.6451	1,705.91	3	8.15E+08
IGSLDNITHVPGGGNKK	853.9622	1,705.91	2	1052510
IGSLDNITHVPGGGNKK	427.487	1,705.92	4	3.26E+07
IGSLDNITHVPGGGNKK	427.4857	1,705.91	4	4857820
IGSLDNITHVPGGGNKK	427.4857	1,705.91	4	897717
IGSLDNITHVPGGGNKK	427.486	1,705.91	4	987470
IGSLDNITHVPGGGNKK	427.4863	1,705.92	4	7266090
IGSTENLK	431.239	860.4635	2	4936660
IGSTENLKHQPGGGK	381.4567	1,521.80	4	428848
KIETHKLTFRENAK	572.3261	1,713.96	3	2728970
KIETHKLTFRENAK	429.4954	1,713.95	4	1560820
KIETHKLTFRENAK	429.4954	1,713.95	4	1179910
KLDLSNVQSK	566.3237	1,130.63	2	6777390
KLDLSNVQSK	377.8845	1,130.63	3	6797930
LDFKDRVQSK	618.3409	1,234.67	2	2709780
LDFKDRVQSK	618.3421	1,234.67	2	4709350
LDFKDRVQSK	618.3406	1,234.67	2	3.65E+07
LDFKDRVQSK	618.3407	1,234.67	2	2.44E+08
LDFKDRVQSK	618.3415	1,234.67	2	2048270
LDFKDRVQSK	618.3406	1,234.67	2	590390
LDFKDRVQSK	412.563	1,234.67	3	4055280
LDFKDRVQSK	412.565	1,234.67	3	1648240
LDFKDRVQSK	412.5636	1,234.67	3	2329710
LDFKDRVQSK	412.5623	1,234.67	3	6494730
LDFKDRVQSK	412.5643	1,234.67	3	621133
LDFKDRVQSK	412.5646	1,234.67	3	631690
LDFKDRVQSK	412.5642	1,234.67	3	605167
LDFKDRVQSK	412.5644	1,234.67	3	1714350
LDFKDRVQSK	412.5627	1,234.67	3	8990510
LDFKDRVQSK	412.5637	1,234.67	3	1405350
LDFKDRVQSKIGSLDNITHVPGGGNK	699.6224	2,794.46	4	2945180
LDFKDRVQSKIGSLDNITHVPGGGNK	699.6266	2,794.48	4	1.04E+07
LDLSNVQSK	502.2763	1,002.54	2	1.49E+08
LDLSNVQSK	502.2764	1,002.54	2	3602480
LDLSNVQSK	502.2762	1,002.54	2	2210260
LTFRENAK	489.7738	977.533	2	1.10E+09
LTFRENAK	489.7716	977.5286	2	1.52E+07
LTFRENAK	489.7725	977.5305	2	2.51E+08
LTFRENAK	489.7747	977.5348	2	2271510
LTFRENAK	489.7745	977.5345	2	925989
LTFRENAK	489.7739	977.5332	2	484169
LTFRENAK	489.7726	977.5306	2	286652
LTFRENAK	489.7739	977.5332	2	467440
LTFRENAK	489.7727	977.5308	2	428820
LTFRENAK	489.7749	977.5352	2	396975
LTFRENAK	489.7744	977.5343	2	313402
LTFRENAK	489.7719	977.5292	2	4153020
LTFRENAK	489.7743	977.5341	2	556436
LTFRENAK	489.7764	977.5382	2	343102
LTFRENAK	489.7726	977.5306	2	325833
LTFRENAK	489.7721	977.5296	2	1304710
LTFRENAK	489.7716	977.5287	2	1926160
LTFRENAK	489.7726	977.5306	2	494865
LTFRENAK	489.7729	977.5312	2	668740
LTFRENAKAK	589.3387	1,176.66	2	1.17E+07
LTFRENAKAKTDHGAEIVYK	573.5607	2,290.21	4	7300470
SEKLDFKDRVQSK	790.4258	1,578.84	2	862065
SEKLDFKDRVQSK	527.2865	1,578.84	3	4558580
SEKLDFKDRVQSK	527.2872	1,578.84	3	5798240

Sequence	Observed m/z	Absolute mass (Da)	Charge	TIC
SEKLDFKDRVQSK	395.7166	1,578.84	4	1045490
SPVVSGDTSPRHLSNVSSTGSIDMVDSPQLATLADEVSASLAK	1,448.05	4,341.14	3	5789350
SPVVSGDTSPRHLSNVSSTGSIDMVDSPQLATLADEVSASLAK	1,086.30	4,341.16	4	3540890
SPVVSGDTSPRHLSNVSSTGSIDMVDSPQLATLADEVSASLAK	869.2347	4,341.14	5	7381630
TDHGAEIVYK	566.7853	1,131.56	2	2.79E+08
TDHGAEIVYK	566.7865	1,131.56	2	5572430
TDHGAEIVYK	378.1934	1,131.56	3	2524120
TDHGAEIVYK	378.1937	1,131.56	3	1684140
TDHGAEIVYK	378.1937	1,131.56	3	1289780
TDHGAEIVYK	378.1925	1,131.56	3	4498190
TDHGAEIVYK	378.1926	1,131.56	3	1391590
TDHGAEIVYK	378.1935	1,131.56	3	1948310
TDHGAEIVYK	378.1924	1,131.56	3	672651
TDHGAEIVYK	566.7853	1,131.56	2	6059200
TDHGAEIVYK	566.7852	1,131.56	2	2.88E+07
TDHGAEIVYK	378.1931	1,131.56	3	3423510
TDHGAEIVYK	566.7856	1,131.56	2	3038160
TDHGAEIVYK	378.1936	1,131.56	3	1510270
TDHGAEIVYK	378.1924	1,131.56	3	5707550
TDHGAEIVYK	566.7869	1,131.56	2	1789910
TDHGAEIVYK	566.7855	1,131.56	2	1.23E+09
TDHGAEIVYK	378.1933	1,131.56	3	2052700
TDHGAEIVYK	566.7878	1,131.56	2	606715
TDHGAEIVYK	378.1934	1,131.56	3	1.32E+07
TDHGAEIVYK	378.1928	1,131.56	3	796246
TDHGAEIVYK	378.194	1,131.56	3	1.24E+09
TDHGAEIVYK	378.1933	1,131.56	3	816200
TDHGAEIVYK	378.1932	1,131.56	3	3017640
TDHGAEIVYK	378.1932	1,131.56	3	1392660
TDHGAEIVYK	378.1932	1,131.56	3	899653
TDHGAEIVYK	566.7892	1,131.56	2	276842
TDHGAEIVYK	566.7862	1,131.56	2	1995230
TDHGAEIVYK	378.1941	1,131.56	3	725129
TDHGAEIVYK	378.1931	1,131.56	3	1147450
TDHGAEIVYK	378.1928	1,131.56	3	793357
TDHGAEIVYK	378.1926	1,131.56	3	4495100
TDHGAEIVYK	566.7894	1,131.56	2	643479
TDHGAEIVYK	566.7849	1,131.56	2	2295400
TDHGAEIVYK	378.1934	1,131.56	3	2985250
TDHGAEIVYK	566.7894	1,131.56	2	619166
TDHGAEIVYK	566.7863	1,131.56	2	1401040
TDHGAEIVYK	378.1931	1,131.56	3	1147240
TDHGAEIVYK	378.1939	1,131.56	3	1067540
TDHGAEIVYK	566.787	1,131.56	2	1621820
TDHGAEIVYK	378.1934	1,131.56	3	828802
TDHGAEIVYK	378.193	1,131.56	3	791990
TDHGAEIVYK	378.1919	1,131.55	3	873625
TDHGAEIVYK	378.1946	1,131.56	3	856211
TDHGAEIVYK	378.1934	1,131.56	3	937579
TDHGAEIVYK	566.788	1,131.56	2	1283420
TDHGAEIVYK	378.1931	1,131.56	3	1210650
TDHGAEIVYK	566.7864	1,131.56	2	222469
TDHGAEIVYK	378.1927	1,131.56	3	656860
TDHGAEIVYK	378.1929	1,131.56	3	674648
TDHGAEIVYK	378.1915	1,131.55	3	646219
TDHGAEIVYK	378.1924	1,131.56	3	868838
TDHGAEIVYK	378.1935	1,131.56	3	652849
TDHGAEIVYK	378.193	1,131.56	3	1234380
TDHGAEIVYK	378.1936	1,131.56	3	1078030
TDHGAEIVYK	378.1939	1,131.56	3	846087
TDHGAEIVYK	378.1937	1,131.56	3	788023
TDHGAEIVYK	378.1931	1,131.56	3	820444
TDHGAEIVYK	378.1934	1,131.56	3	4.24E+08
TDHGAEIVYK	378.1934	1,131.56	3	7208060
TDHGAEIVYK	566.786	1,131.56	2	1689280
TDHGAEIVYK	378.1951	1,131.56	3	1187870
TDHGAEIVYK	378.1952	1,131.56	3	700429
TDHGAEIVYK	378.1929	1,131.56	3	805639
TDHGAEIVYK	378.1937	1,131.56	3	994250
TDHGAEIVYK	378.1927	1,131.56	3	648024
TDHGAEIVYK	566.7864	1,131.56	2	795048
TDHGAEIVYK	378.1926	1,131.56	3	675877
TDHGAEIVYK	566.7859	1,131.56	2	355248
TDHGAEIVYKSPVVSGDTSPRHLSNVSSTGSIDMVDSPQLATLADEVSASLAK	1,091.95	5,454.70	5	4.77E+07
TDHGAEIVYKSPVVSGDTSPRHLSNVSSTGSIDMVDSPQLATLADEVSASLAK	907.4586	5,438.71	6	5919710
TDHGAEIVYKSPVVSGDTSPRHLSNVSSTGSIDMVDSPQLATLADEVSASLAK	1,088.75	5,438.72	5	600274

Supplementary Fig. 3 | Details of the peptides detected (shown in Fig. 2c, orange) by enzymatic digestion of the trypsin protected core (Fig. 2b) of heparin-free 2N4R tau fibrils.

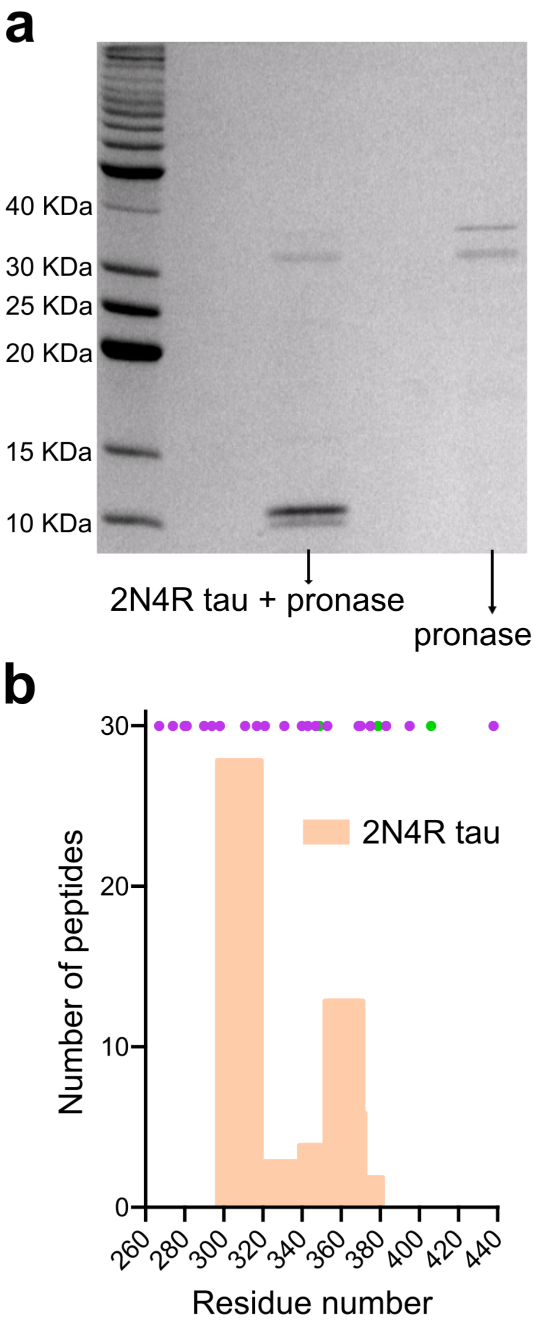

c

Sequence	m/z	mass (Da)	Charge	TIC
HVPGGGSVQIVYKPVDLSK	660.7014	1,979.08	3	9422240
HVPGGGSVQIVYKPVDLSK	660.7024	1,979.09	3	2133380
HVPGGGSVQIVYKPVDLSK	660.702	1,979.08	3	2.22E+07
HVPGGGSVQIVYKPVDLSK	660.7023	1,979.09	3	2399590
HVPGGGSVQIVYKPVDLSK	660.702	1,979.08	3	3936100
HVPGGGSVQIVYKPVDLSK	660.7021	1,979.08	3	544054
HVPGGGSVQIVYKPVDLSK	660.7022	1,979.08	3	703770
HVPGGGSVQIVYKPVDLSK	660.7018	1,979.08	3	736070
HVPGGGSVQIVYKPVDLSK	660.7022	1,979.08	3	1097150
HVPGGGSVQIVYKPVDLSK	660.702	1,979.08	3	548185
HVPGGGSVQIVYKPVDLSK	660.7019	1,979.08	3	308571
HVPGGGSVQIVYKPVDLSK	495.7784	1,979.08	4	611012
HVPGGGSVQIVYKPVDLSK	495.7787	1,979.09	4	1431220
HVPGGGSVQIVYKPVDLSK	660.7021	1,979.08	3	548642
HVPGGGSVQIVYKPVDLSK	660.7021	1,979.08	3	1309960
HVPGGGSVQIVYKPVDLSK	660.7024	1,979.09	3	429216
HVPGGGSVQIVYKPVDLSK	660.7022	1,979.08	3	452406
HVPGGGSVQIVYKPVDLSK	495.7783	1,979.08	4	2.26E+07
HVPGGGSVQIVYKPVDLSK	990.5488	1,979.08	2	362834
HVPGGGSVQIVYKPVDLSK	660.7023	1,979.09	3	1530500
HVPGGGSVQIVYKPVDLSK	660.7019	1,979.08	3	456421
HVPGGGSVQIVYKPVDLSK	495.7787	1,979.09	4	274176
HVPGGGSVQIVYKPVDLSK	660.7033	1,979.09	3	325718
HVPGGGSVQIVYKPVDLSK	660.7023	1,979.09	3	264003
HVPGGGSVQIVYKPVDLSK	660.7021	1,979.08	3	468775
HVPGGGSVQIVYKPVDLSK	660.7023	1,979.09	3	283906
HVPGGGSVQIVYKPVDLSK	660.7018	1,979.08	3	309846
HVPGGGSVQIVYKPVDLSK	660.7022	1,979.08	3	234939
VTSKCGSLGNIHHKPGGGQVEVK	478.6541	2,388.23	5	334355
VTSKCGSLGNIHHKPGGGQVEVK	598.0657	2,388.23	4	952181
CGSLGNIHHKPGGGQVEVK	658.671	1,972.99	3	897374
SEKLDFKDR	379.8681	1,136.58	3	1609850
SEKLDFKDR	379.868	1,136.58	3	707734
SEKLDFKDR	379.8681	1,136.58	3	365798
SEKLDFKDR	569.2987	1,136.58	2	531358
IGSLDNITHVPGGGNK	526.9463	1,577.82	3	2020820
IGSLDNITHVPGGGNK	526.9461	1,577.82	3	9411440
IGSLDNITHVPGGGNK	789.9155	1,577.82	2	1135550
IGSLDNITHVPGGGNK	526.9463	1,577.82	3	328157
IGSLDNITHVPGGGNK	789.9142	1,577.81	2	246760
IGSLDNITHVPGGGNK	789.9131	1,577.81	2	315806
IGSLDNITHVPGGGNK	526.9461	1,577.82	3	308279
IGSLDNITHVPGGGNK	526.9463	1,577.82	3	1114760
IGSLDNITHVPGGGNKK	569.6443	1,705.91	3	1.06E+08
IGSLDNITHVPGGGNKK	569.6446	1,705.91	3	441671
IGSLDNITHVPGGGNKK	427.4852	1,705.91	4	1.64E+08
IGSLDNITHVPGGGNKK	427.4853	1,705.91	4	2935320
IGSLDNITHVPGGGNKK	427.4853	1,705.91	4	443302
KIETHKLTFR	424.9188	1,271.73	3	267016
IETHKLTFR	382.2207	1,143.64	3	181627

Supplementary Fig. 4 | Pronase-resistant core of heparin-free 2N4R tau fibrils. a, SDS-PAGE gel of pronase-digested tau fibrils formed in the absence of heparin. The pronase band is indicated in a separate lane. The result was reproducible for three independently performed experiments. **b,** Number of peptides detected from the enzymatic digestion of the tau band observed in SDS-PAGE in (a). The position of lysine and arginine residues in 2N4R tau are marked with purple and green dots, respectively. **c,** Details of the peptides detected as shown in (b).

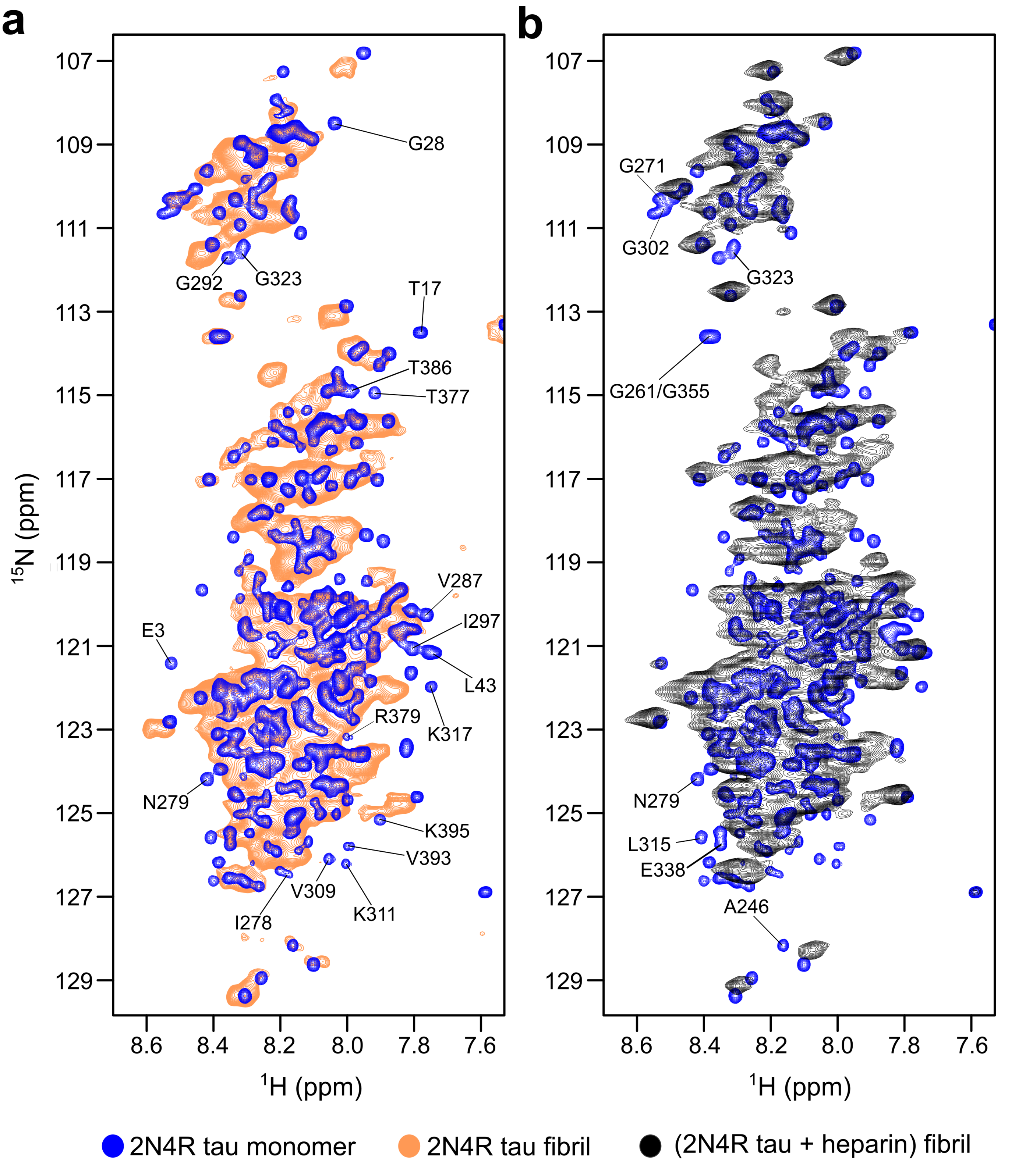

Supplementary Fig. 5 | Superposition of the ^{1}H-^{15}N HSQC spectrum of monomeric 2N4R tau (blue) with ^{1}H-^{15}N INEPT spectra of heparin-free 2N4R fibrils (orange; left) and heparin-induced fibrils of 2N4R tau (black; right).

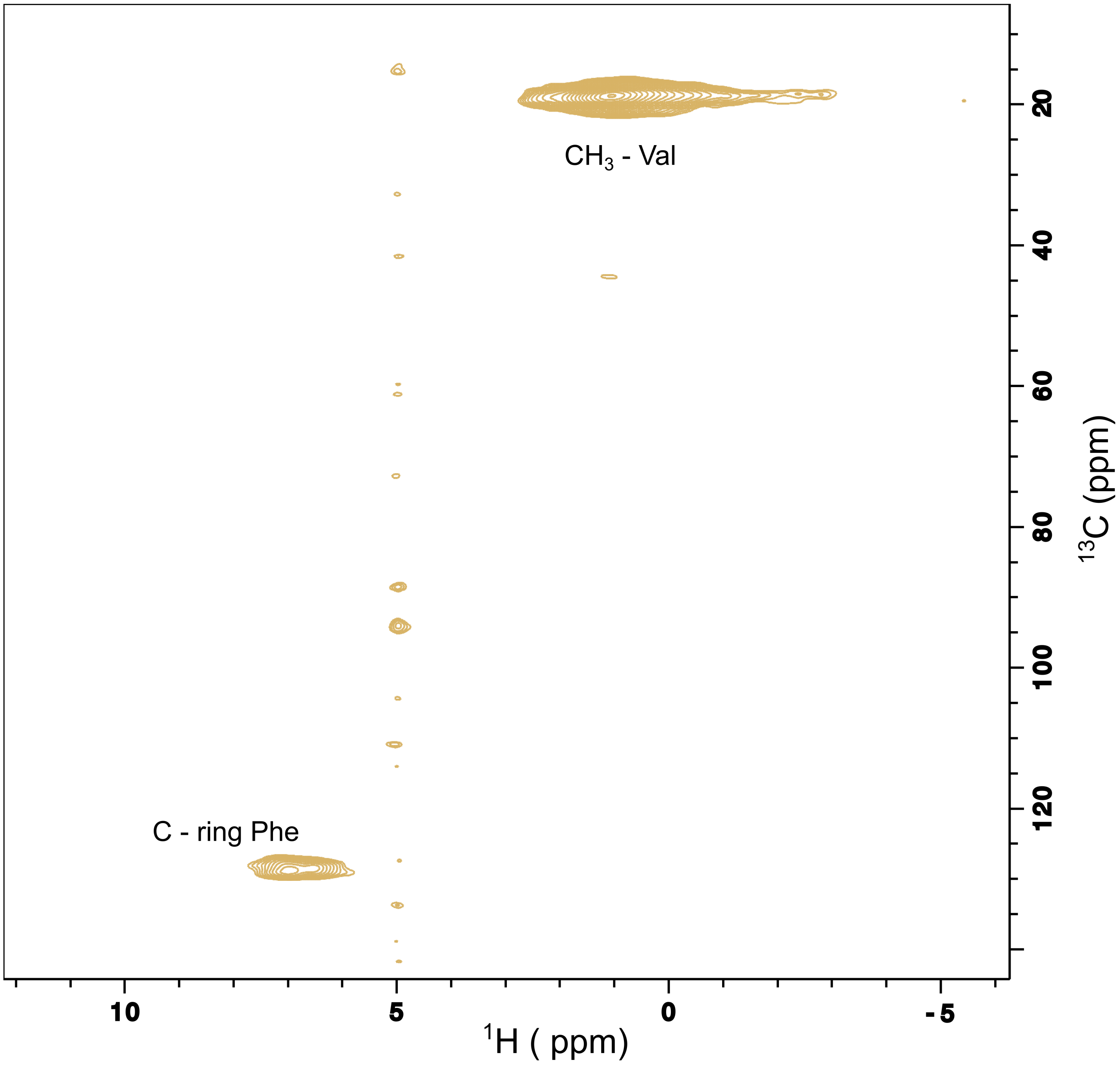

Supplementary Fig. 6 | 2D hCH spectra of heparin-free fibrils of $^{13}C_{\gamma}$ valine, ^{13}C-ring phenylalanine-labeled 2N4R tau.

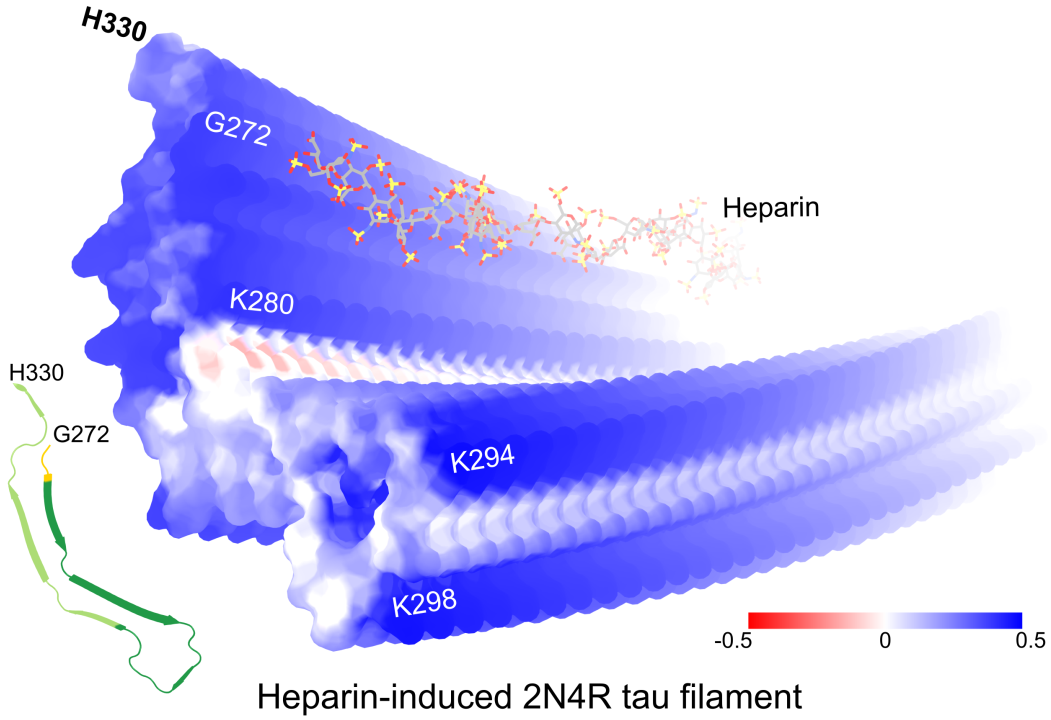

Supplementary Fig. 7 | Electrostatic surface potential of the heparin-induced 2N4R tau fibrils (snake form; PDB code: 6QJH). Positively and negatively charged residues are shown in blue and red, respectively. A molecule of heparin is displayed to illustrate the presence of heparin on the surface of the fibril.
