# Supplementary material for: Co-factor-free aggregation of tau into seeding-competent RNA-sequestering amyloid fibrils
Source: Nat Commun. 2021 Jul 9;12:4231. doi: 10.1038/s41467-021-24362-8 (PMC8270918; doi:10.1038/s41467-021-24362-8)
Supplement: Supplementary file 3 — Reporting summary [file 41467_2021_24362_MOESM3_ESM.pdf]

nature research

Corresponding author(s): Markus Zweckstetter

Last updated by author(s): Jun 6, 2021

Reporting Summary

Nature Research wishes to improve the reproducibility of the work that we publish. This form provides structure for consistency and transparency in reporting. For further information on Nature Research policies, see our Editorial Policies and the Editorial Policy Checklist.

Statistics

For all statistical analyses, confirm that the following items are present in the figure legend, table legend, main text, or Methods section.

n/a	Confirmed	
[ ]	[x]	The exact sample size (n) for each experimental group/condition, given as a discrete number and unit of measurement
[ ]	[x]	A statement on whether measurements were taken from distinct samples or whether the same sample was measured repeatedly
[ ]	[x]	The statistical test(s) used AND whether they are one- or two-sided<br>*Only common tests should be described solely by name; describe more complex techniques in the Methods section.*
[x]	[ ]	A description of all covariates tested
[x]	[ ]	A description of any assumptions or corrections, such as tests of normality and adjustment for multiple comparisons
[ ]	[x]	A full description of the statistical parameters including central tendency (e.g. means) or other basic estimates (e.g. regression coefficient) AND variation (e.g. standard deviation) or associated estimates of uncertainty (e.g. confidence intervals)
[ ]	[x]	For null hypothesis testing, the test statistic (e.g. F, t, r) with confidence intervals, effect sizes, degrees of freedom and P value noted<br>*Give P values as exact values whenever suitable.*
[x]	[ ]	For Bayesian analysis, information on the choice of priors and Markov chain Monte Carlo settings
[x]	[ ]	For hierarchical and complex designs, identification of the appropriate level for tests and full reporting of outcomes
[x]	[ ]	Estimates of effect sizes (e.g. Cohen's d, Pearson's r), indicating how they were calculated

Our web collection on statistics for biologists contains articles on many of the points above.

Software and code

Policy information about availability of computer code

Data collection	NMR data were acquired using Topspin 3.6.2 and 4.0.1; ThT fluorescence data were collected using Spark control software v 2.2 by Tecan, CD data were collected using Chirascan software v 4.5
Data analysis	NMR data were processed and analyzed with Topspin 3.6.2 and CCPNMR 2.4.2, respectively. Graphpad prism v 9 were used for fitting data as well as for statistical analysis. UCSF Chimera (v 1.14.0) was used to analyze the surface electrostatic potential of tau fibril from CBD and heparin-induced 2N4R tau fibril. CDNN sofware v 2 (Chirascan) was used to analyze the CD spectra of heparin-free 2N4R tau fibril. Scaffold 4 software was used to analyze the MS data.

For manuscripts utilizing custom algorithms or software that are central to the research but not yet described in published literature, software must be made available to editors and reviewers. We strongly encourage code deposition in a community repository (e.g. GitHub). See the Nature Research guidelines for submitting code & software for further information.

Data

Policy information about availability of data

All manuscripts must include a data availability statement. This statement should provide the following information, where applicable:

- Accession codes, unique identifiers, or web links for publicly available datasets
- A list of figures that have associated raw data
- A description of any restrictions on data availability

All the PDB codes (6TJO, 6QJH) cited in this paper are available in the protein data bank web server. Source data of Fig. 5;6 and Supplementary Fig. 1 is provided with this paper as an excel file. Data that support the findings of this study are available from the corresponding authors upon reasonable request.

Field-specific reporting

Please select the one below that is the best fit for your research. If you are not sure, read the appropriate sections before making your selection.

☒ Life sciences ☐ Behavioural & social sciences ☐ Ecological, evolutionary & environmental sciences

For a reference copy of the document with all sections, see nature.com/documents/nr-reporting-summary-flat.pdf

Life sciences study design

All studies must disclose on these points even when the disclosure is negative.

Sample size	The ThT fluorescence assay of tau in presence/absence of heparin as well as the in-vitro and in-cell seeding assay was performed with three independent samples. The interaction of tau fibrils in presence/absence of heparin with polyU, polyA and tRNA (both in 10 mM and 100 mM KCl) was performed with three independtly prepared samples. We used a sample size of 3 for each experiments to ensure the data is statistically relevant.
Data exclusions	No data have been excluded while reporting this study.
Replication	The ThT fluorescence assay of tau in absence of heparin has been performed up to 35 times with 7 different batches of protein. In all cases the data were reproducible. The micrographs of 2N4R tau fibrils in presence/absence of heparin was recorded for 10 samples and in all cases the data were reproducible. The trypsin digestion of both 2N4R tau fibrils is repeated 3 times as well as the pronase digestion of heparin-free 2N4R tau fibrils was repeated 3 times. The in-vivo seeding of tau in HEK293T biosensor cells was repeated 3 times. In all cases the data were reproducible.
Randomization	Randomization was not relevant to this study, because we are not working with any particular population and our experiments can be repeated.
Blinding	Blinding was not relevant to this study, because decision-making has no impact on the experiment and there is no risk of bias.

Reporting for specific materials, systems and methods

We require information from authors about some types of materials, experimental systems and methods used in many studies. Here, indicate whether each material, system or method listed is relevant to your study. If you are not sure if a list item applies to your research, read the appropriate section before selecting a response.

Materials & experimental systems

n/a	Involved in the study	
☒	☐	Antibodies
☐	☒	Eukaryotic cell lines
☒	☐	Palaeontology and archaeology
☒	☐	Animals and other organisms
☒	☐	Human research participants
☒	☐	Clinical data
☒	☐	Dual use research of concern

Methods

n/a	Involved in the study	
☒	☐	ChIP-seq
☒	☐	Flow cytometry
☒	☐	MRI-based neuroimaging

Eukaryotic cell lines

Policy information about cell lines

Cell line source(s)	HEK293 Tau-GFP (doi: https://doi.org/10.1101/2020.06.26.173070). These cells were engineered to stably express the human tau repeat domain (RD) with point mutations P301L/V337M and a carboxyterminal GFP-tag (thereafter termed TauRD-GFP).
Authentication	HEK293 authenticated at ECACC (#85120602)
Mycoplasma contamination	Cells tested negative for mycoplasma contamination
Commonly misidentified lines (See ICLAC register)	None
